# Postoperative lordosis distribution index, patient reported outcome measures, and revision surgery following transforaminal lumbar interbody fusion

**DOI:** 10.1016/j.wnsx.2023.100251

**Published:** 2023-12-12

**Authors:** Anders Schack, Tanvir Johanning Bari, Martin Gehrchen, Benny Dahl, Rachid Bech-Azeddine

**Affiliations:** a Copenhagen Spine Research Unit (CSRU), Rigshospitalet, Glostrup, Valdemar Hansens Vej 17, 2600, Glostrup, Denmark; b Spine Unit, Department of Orthopedic Surgery, Rigshospitalet, Copenhagen University Hospital, Blegdamsvej 9, 2100, Copenhagen, Denmark; c Department of Clinical Medicine, University of Copenhagen, Copenhagen, Denmark

**Keywords:** Lordosis distribution index, Patient reported outcomes, Spine fusion, Oswestry disability index, Lumbar spine

## Abstract

**Objective:**

Lordosis Distribution Index (LDI) is a new radiographic parameter associated with postoperative residual symptoms in patients undergoing Transforaminal Lumbar Interbody Fusion (TLIF). Recently, it has been applied on patients undergoing instrumented spine surgery, however not correlated to Patient Related Outcome Measures (PROMs). This study investigates whether the obtained the postoperative LDI after TLIF surgery correlates with the clinical outcome measured with PROMs.

**Methods:**

This study was based on prospectively obtained data in patients undergoing TLIF throughout 2017 at a Danish university hospital. Medical records and the DaneSpine Database were accessed to obtain preoperative, operative and follow-up data. Primary outcome was Oswestry Disability Index (ODI) 12 months postoperatively. Secondary outcomes included revision rate and additional PROMs.

**Results:**

126 patients were included. 70 patients were classified with normolordosis (56 %), 42 hypolordosis (33 %) and 14 hyperlordosis (11 %). All groups experienced significant radiological changes undergoing surgery. Average reduction in ODI at 12 months postoperatively was −15.3 (±20.0). Minimally clinical important difference was achieved in 68 patients (54.0 %). No significant difference in PROMs between LDI-groups was observed in unadjusted or adjusted analyses. Revision surgery was performed in 8 patients with normolordosis (11.4 %), 7 hypolordosis (16.7 %) and 4 hyperlordosis (28.6 %).

**Conclusions:**

We found no significant correlation between postoperative LDI subgroups of normolordotic, hypo- or hyperlordotic patients and the clinical outcome of posterolateral fusion and TLIF surgery. A trend towards lower rate of revision surgery in the normolordotic group compared to the hypo- and hyperlordotic group was observed.

## 1 Introduction

Degenerative lumbar spine diseases are among the most frequent reasons for decreased health related quality of life.^1^, ^2^, ^3^ Lumbar degenerative pathologies can cause low back pain with or without radicular pain, and fusion surgery in the context of explicit degenerative changes on MRI can be utilized for treatment in select cases.^4^^,^^5^ Posterolateral fusion with pedicle screws (PLF) combined with transforaminal lumbar interbody fusion (TLIF) has proven to produce satisfactory clinical results, obtaining a circumferential arthrodesis with a posterior approach and minimal affection of neural elements.^6^^,^^7^ The aim of PLF with TLIF is to reduce pain through reduced motion and load on the degenerative level. However, symptoms can reside after surgery despite a technically adequate surgical procedure and no postoperative complications. In patients with 1–3 levels of lumbar degenerative disease undergoing fusion, the postoperative distribution of lordosis is not a fully explored aspect and could potentially explain residual pain despite a surgically successful procedure, especially since iatrogenic loss of lordosis has been proposed as a reason for unsuccessful surgery.^8^, ^9^, ^10^

**Fig. 1:**
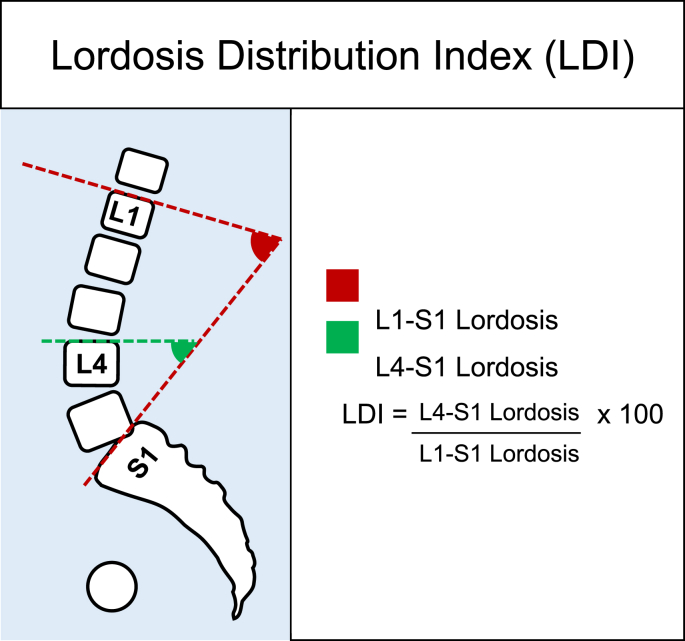
Lordosis distribution index (LDI), defined as the ratio between the lower lordosis (L4–S1) and the global lordosis. By nature, this should expose the increasing lordosis towards the lower spinal segments.

It has been described that the mismatch between the pelvic incidence and the lumbar lordosis (PI-LL mismatch) after lumbar spinal fusion may increase the risk of adjacent level degeneration.^11^ However, the Pl-LL concept mainly covers the magnitude of lordosis and not how the lordosis should be distributed. More recently, the lordosis distribution index (LDI) has been suggested as a more precise quantification of lumbar lordosis.^12^, ^13^, ^14^ LDI was proposed in relation to surgery for adult spinal deformity surgery as a radiographic aim to quantify the distribution of the lumbar lordosis in an increasing fashion from the lower segment of the lumbar lordosis (L4-S1) to a lesser extent at the top of the lordosis. The index describes the ratio between the lordosis at L4-LS1 and the lordosis at L1-S1, and spans from 0 to 100 % (Fig. 1). In a recent study, normal values of LDI were described as 50–80 %. LDI <50 % implies hypolordotic maldistribution, and LDI >80 % suggests hyperlordotic maldistribution.^12^

The LDI has been proposed as a supplement to the known radiographic concept of the magnitude of lordosis using the PI, LL and the PI-LL mismatch, which has been associated with postoperative residual symptoms such as low back pain, lower extremity pain and numbness in patients undergoing TLIF.^8^^,^^15^

Several radiographical parameters have been correlated with pain and disability. To assess the clinical implications of postoperative LDI, Patient Reported Outcome Measures (PROMs) were chosen as the primary outcome of the present study.^16^^,^^17^ To classify meaningful, clinical change for the individual patient, Minimal Clinical Important Difference (MCID) was evaluated, and PROMs were collected prospectively as part of the national DaneSpine database for patients undergoing spine surgery.

Few studies have focused on LDI and the outcome after lumbar instrumented fusion^12^^,^^14^^,^^18^ and to our knowledge no studies have included a PROM as an outcome variable. The purpose of the present study was to assess whether postoperative LDI after TLIF surgery correlates with the clinical outcome measured with PROMs at one year follow-up.

## 2 Materials and methods

### 2.1 Study design

We conducted a retrospective cohort study based on prospectively obtained data from the DaneSpine Database, which is a national spine surgery database for prospective collection of demographics as well as pre- and postoperative PROMs.

From the DaneSpine Database, patients undergoing short segment PLF with TLIF surgery at our department during a 1-year period from January 1st, 2017, to December 31st, 2017, were identified.

Inclusion criteria for the current study were availability of preoperative and postoperative radiographs presenting 1) femoral heads, 2) sacral endplate and 3) all lumbar vertebras. Furthermore, patients were included when having filled out the DaneSpine questionnaire with PROM data preoperatively and at 12 months postoperatively.

All patients undergoing surgery were eligible for inclusion.

The study was conducted as a quality control study and approved by the local hospital committee. As a retrospective follow-up study informed consent was not required for approval. An IRB approval number was not provided for this study as a standard, since it as a quality control study.

### 2.2 Patient sample

Included patients underwent fusion surgery of 1–3 motion segment levels for degenerative lumbar spine pathologies, comprising one or a combination of the following conditions: anterolisthesis, retrolisthesis, degenerative disc disease, foraminal stenosis, lumbar stenosis with facet joint arthropathy, spondylosis, and recurrent disc herniation. The clinical indication for fusion was persistent LBP ≥ six months and/or radicular symptoms due to foraminal stenosis resistant to conservative treatment. The surgery consisted of decompression of the stenosis, posterolateral fusion with pedicle screws and placement of a TLIF at each level of fusion.

Charleston Comorbidity Index (CCI),^19^^,^^20^ surgical data and revisions were acquired from electronic medical records. From DaneSpine, patient characteristics, back and leg pain visual analog scale (VAS 0 to 100),^21^ Oswestry Disability Index (ODI)^22^ and European Quality of Life – 5 Dimensions Questionnaire (EQ-5D-3L)^23^ was obtained preoperatively and 12 months postoperatively.

MCID for the PROM data were defined as ODI-score of 10,^24^ VAS-Back of 18,^24^ VAS-Leg of 20^17^ and EQ-5D of 0.19.^25^^,^^26^

Radiographic examinations were obtained preoperatively and 3 months post-surgery and were assessed using the online imaging system KEOPS (SMAIO, Lyon, France).^27^ LDI was assessed as ratio between 1) the upper endplate of L4 to S1 2) the global lordosis, see Fig. 1.

Primary outcome was ODI at 12 months postoperatively.

Secondary outcomes were revisions and the additional PROMs: VAS-back pain, VAS-leg pain and EQ-5D-3L.

### 2.3 Statistical analyses

Patients were categorized by the calculated postoperative LDI as: hypolordosis (LDI<50), normal (LDI 50–80), or hyperlordosis (LDI>80). Categorical data were presented as proportions (%). Age, BMI, and CCI-score were categorized into subgroups to facilitate interpretation. Continuous data were assessed for normal distribution by visual assessment of QQ-plots and presented as means with standard deviation (SD) and medians with interquartile range (IQR). Unadjusted analyses were students *t*-test or Mann–Whitney *U* test for continuous data. *χ*2 test or Fisher's exact test was used for categorical data. Analysis of variance (ANOVA) was performed when comparing continuous data of the three LDI groups, followed by a pairwise comparison of normolordotic and respectively hypo- and hyperlordotic groups.Multivariate logistic regression was performed by forced entry method, each model applied by itself, with MCID for ODI as the dependent variable. LDI group was considered the independent variable. Additional variables were prespecified based on clinical hypothesis. Results are presented as odds ratio (OR) with 95 % confidence intervals.

Finally, the change in ODI was examined by applying a multivariate analysis of variance (MANOVA), comparing all three groups in three separate models. The *p*-value of LDI-group as an independent variable is reported. Both multivariate analyses were examined for interaction of independent variables.

### 2.4 Multivariate analyses were repeated with MCID for EQ-5D as the dependent variable

Statistical analyses were performed using SPSS statistical software, v28.0.0, (IBM corporation, USA). Two-sided *p*-values <0.05 were considered statistically significant.

## 3 Results

In total, 260 patients undergoing instrumented spinal fusion with TLIF were identified. Of these, 150 patients (58 %) had fulfilled the DaneSpine questionnaire 12 months after surgery, of which 24 (16 %) patients had insufficient postoperative radiographical imaging and were excluded.

Thus, 126 patients were included for final analyses. Table 1 shows patient characteristics. Of all included patients, 82 (65 %) were female, mean age ±SD at time of surgery was 57 ± 12. Mean BMI was 28.2 ± 5 (SD) and median CCI was 2 (IQR: 1–3). No significant difference was found between included patients and patients excluded due to lack of imaging.

**Table 1:** Demographic distribution between groups. Abbreviations: LDI: Lordosis Distribution Index, BMI: Body Mass Index, CCI: Charleston Comorbidity index.

LDI		Normolordosis (*n* = 70)		Hypolordosis (*n* = 42)		Hyperlordosis (*n* = 14)		p
Female		39	55.7 %	35	83.3 %	8	57.1 %	0.01
Age	0–60	44	62.9 %	23	54.8 %	8	57.1 %	0.833
	61–70	17	24.3 %	13	31.0 %	4	28.6 %	
	71+	9	12.9 %	6	14,3 %	2	14.3 %	
BMI	<25	11	19.3 %	14	36.8 %	1	8.3 %	0.144
	25–30	28	49.1 %	9	23.7 %	7	58.3 %	
	>30	18	31.6 %	15	39.4 %	4	33.4 %	
Smoking	Yes	14	20 %	13	31 %	3	21 %	0.402
CCI	0	20	28.6 %	7	16.7 %	2	14.3 %	0.469
	1–2	27	38.6 %	21	50.0 %	8	57.1 %	
	3+	23	32.9 %	14	33.3 %	4	28.6 %	
Surgical indication	Stenosis	2	2.9 %	3	7.1 %	1	7.1 %	0.000
	Spondylotic radiculopathy	20	28.6 %	5	11.9 %	6	42.9 %	
	Spondylosis	5	7.1 %	3	7.1 %	0	0.0 %	
	Retrolisthesis	1	1.4 %	0	0.0 %	0	0.0 %	
	Disc herniation	0	0.0 %	1	2.4 %	4	28.6 %	
	Degenerative Disc Disease	18	25.7 %	3	7.1 %	0	0.0 %	
	Anterolisthesis	24	34.3 %	27	64.3 %	3	21.4 %	
Revision surgery		8	11.4 %	7	16.7 %	4	28.6 %	0.332

Revision surgery within 12 months of index procedure was performed in 8 normolordosis patients (11.4 %), 7 hypolordosis patients (16.7 %) and 4 hyperlordosis patients (28.6 %).

### 3.1 Radiographical outcomes

**Fig. 2:**
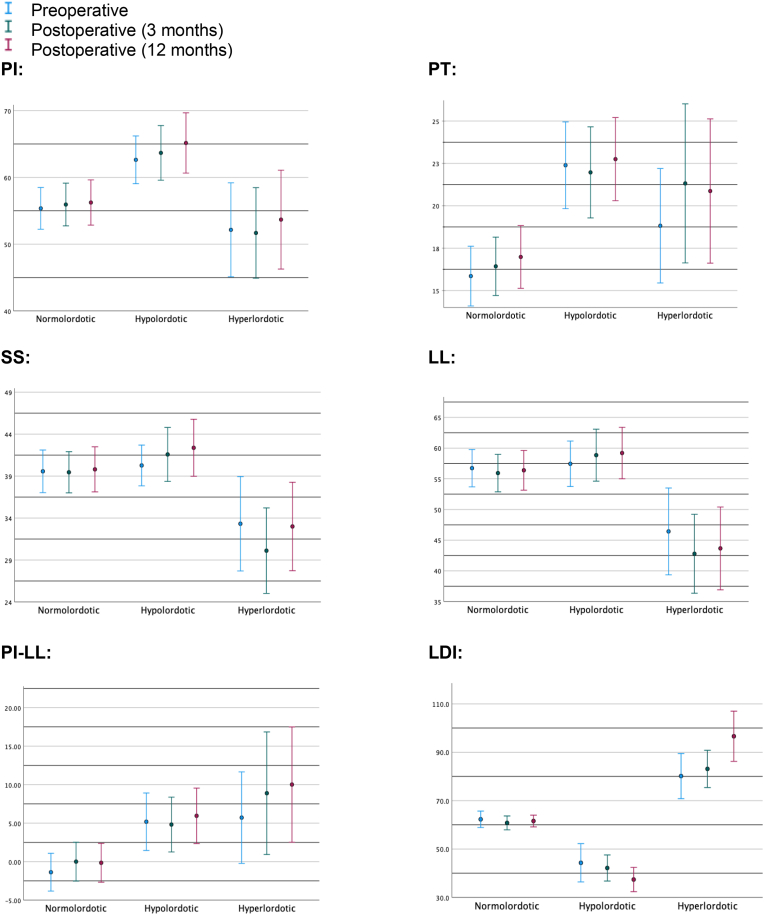
Distribution of radiographical parameters preoperatively, at 3 months postoperatively and 12 months postoperatively. Absolute values are depicted, except from PI-LL, where the difference between the parameters is visualized. Abbreviations: PI: Pelvic Incidence, PT: Pelvic Tilt, SS: Sacral Slope, LL: Lumbar Lordosis, LDI: Lordosis distribution index.

The values of the radiographical parameters (PI, PT, SS, LL, PI-LL and LDI) preoperatively, 3 months after surgery and 12 months after surgery are shown in Fig. 2.

The degree of change from pre-to postoperatively in Pelvic Tilt (PT) and Sacral Slope (SS), but not in lordosis (LL) or LDI, differed significantly when stratified according to the individual LDI group (normo-, hypo- or hyperlordotic, Table 2). The change in Pelvic Tilt (PT) thus was 0.59 (SD = 3.9) for the normolordotic group compared to −0.45 (SD = 4.1) for the hypo- and 3.0 (SD = 4.5) for the hyperlordotic group (ANOVA, *p* = 0.024). The change in Sacral Slope (SS) was −0.15 (SD = 5.4) in the normolordotic group, 1.4 in the hypo- (SD = 7.5) and −3.8 (SD = 7.7) in the hyperlordotic group (ANOVA, *p* = 0.034). LDI tended to exaggerate after surgery, i.e., normolordotic patients stayed normolordotic LDI = −1.4 (SD = 10.7), whereas hypolordotic, 2.0 (SD = 19.0) and hyperlordotic 3.2 (SD = 20.6) progress in their respective directions, although the change was not significantly different (*p* = 0.593)

**Table 2:** Radiographical outcomes of surgery. The table shows the difference between 3 months postoperatively and preoperatively. Mean change (SD). Abbreviations: LDI: Lordosis distribution Index, SD: standard derivation.

LDI-group	Normolordotic	Hypolordotic	Hyperlordotic	p
ΔPelvic Tilt	0.59 (3.9)	−0.45 (4.1)	3.0 (4.5)	0.024
ΔLordosis	−0.81 (7.2)	1.47 (9.4)	−4.2 (10.2)	0.078
ΔSacral Slope	−0.15 (5.4)	1.4 (7.5)	−3.8 (7.7)	0.034
ΔLDI	−1.4 (10.7)	−2.0 (19.0)	3.2 (20.6)	0.593

### 3.2 Patient related outcome measures

Considering the overall effect of TLIF surgery, an improvement in clinical outcome was observed (Table 3) (see Table 4).

**Table 3:** Patient Related Outcome Measures of the entire patient population preoperatively and 12 months postoperatively, mean (SD). Furthermore, the difference between 12 months follow-up and preoperatively and the number of patients reached MCID is seen. Abbreviations: MCID: Minimal Clinical Important Difference, ODI: Oswestry Disability Index, VAS: Visual analog Scale, EQ-5D: European Quality of Life – 5 Dimensions Questionnaire.

	Preoperatively	12 months	Difference (12 months)	MCID (12 months)
ODI	46.3 (15.3)	30.1 (20.2)	−15.3 (20.0)	68 (54.0 %)
VAS**-**back	69.9 (20.3)	39.8 (30.1)	−30.5 (32.5)	75 (59.5 %)
VAS-legs	60.5 (26.7)	31.4 (32.7)	−28.0 (38.2)	65 (51.6 %)
EQ-5D	0.46 (0.23)	0.65 (0.26)	0.19 (0.27)	57 (38.0 %)

**Table 4:** Patient Related Outcome Measures for the three individual post-operative LDI-groups as mean (SD) and absolute numbers (percentage).

LDI	Normolordotic	Hypolordotic	Hyperlordotic	p	p*	p^#^
ΔODI	−14.2 (17.9)	−16.9 (21.1)	−15.4 (26.4)	0.819	0.744	0.914
ODI MCID	37 (52.9 %)	24 (57.1 %)	7 (50.0 %)	0.863	0.659	0.845
ΔVAS-legs	−28.8 (39.2)	−29.1 (37.0)	−20.9 (39.4)	0.793	0.994	0.529
VAS-legs MCID	36 (51.4 %)	19 (45.2 %)	6 (42.9 %)	0.742	0.526	0.558
ΔVAS-back	−29.5 (31.8)	−30.1 (28.9)	−36.5 (33.8)	0.772	0.951	0.420
VAS-back MCID	41 (58.6 %)	26 (61.9 %)	8 (57.1 %)	0.924	0.728	0.921
ΔEQ-5D	0.18 (0.30)	0.17 (0.26)	0.25 (0.22)	0.636	0.968	0.361
MCID EQ-5D	22 (37.3 %)	18 (46.2 %)	6 (50.0 %)	0.576	0.382	0.411

**p:** ANOVA, all three groups.

p*: Pairwise test. Mann–Whitney U, normolordotic compared to hypolordotic.

**p**^**#**^**:** Pairwise test. Mann–Whitney U, normolordotic compared to hyperlordotic.

Abbreviations: MCID: Minimal Clinical Important Difference, ODI: Oswestry Disability Index, VAS: Visual analog Scale, EQ-5D: European Quality of Life – 5 Dimensions Questionnaire.

**Fig. 3:**
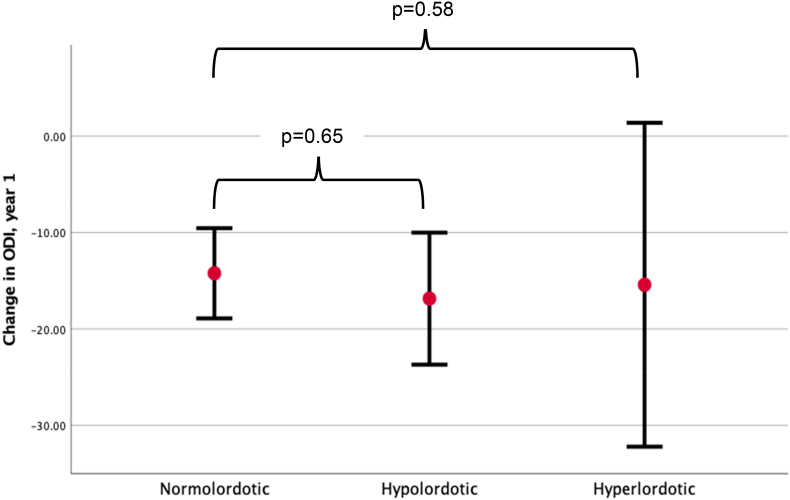
Development in ODI-score from before surgery to 12 months after surgery, grouped by post-operative LDI-group. Abbreviations: ODI: Oswestry Disability Index. LDI: Lordosis Distribution Index.

The average reduction in the primary outcome, ODI 12 months postoperatively, was −15.3 (±20.0), and MCID was achieved in 68 patients (54.0 %, Fig. 3),

A VAS-back pain reduction of −30.5 (±32.5), VAS-leg pain reduction of −28.0 (±38.2) and an increase in EQ-5D of 0.18 (±0.27) was observed, corresponding to that MCID was obtained in VAS-back pain in 75 (59.5 %), VAS-leg pain in 65 (51.6 %) and EQ-5D in 78 patients (61.9 %).

The individual PROMs of the three LDI-groups at 12 months after surgery are shown in Table 3. ANOVA analysis of all three groups revealed no significant differences between groups. Nor did a following pairwise analysis of normolordotic opposed to hypolordotic, and normolordotic opposed to hyperlordotic show any significant differences in PROMs.

The adjusted analyses did not show a significant difference between the LDI groups. A logistic regression, with MCID of ODI as dependent variable is shown in Table 5. With normolordosis as reference, change of LDI-group did not affect the risk of achieving MCID. This was consistently the case in the stepwise adjusted analyses.

**Table 5:** Logistic regression on achieved MCID of ODI 12 months after surgery with normolordotic distribution as reference.

Postioperative LDI group	OR (95 % CI)	p
Model 1		
Normolordotic	–	–
Hypolordotic	1.2 (0.6–2.6)	0.66
Hyperlordotic	0.9 (0.3–2.8)	0.85
**Model 2**		
Normolordotic	–	–
Hypolordotic	1.2 (0.5–2.8)	0.62
Hyperlordotic	0.9 (0.3–2.9)	0.88
**Model 3**		
Normolordotic	–	–
Hypolordotic	0.4 (0.1–1.4)	0.17
Hyperlordotic	1.1 (0.2–7.0)	0.90

Model 1: Unadjusted.

Model 2: Adjusted for sex and age.

Model 3: Adjusted for sex, age, smoking status, CCI, BMI and surgical indication.

Abbreviations: LDI: Lordosis Distribution Index, OR: Odds Ratio, CI: Confidence interval, MCID: Minimal Clinical Important Difference, ODI: Oswestry disability Index, CCI: Charleston Comorbidity Index, BMI: Body Mass Index.

Finally, a MANOVA analyses examined the effect on the absolute change in ODI, and the reported *p*-values are shown in Table 6. There was no significant effect of LDI group on ODI in neither the unadjusted (*p* = 0.65), adjusted for sex and age (*p* = 0.83) nor when adjusting for sex, age, surgical indication, CCI and BMI (*p* = 0.83).

**Table 6:** MANOVA analysis with LDI as independent variable, on change in ODI from preoperatively to 12 months after surgery.

Model:	p
1	0.65
2	0.83
3	0.82

Model 1: Unadjusted.

Model 2: Adjusted for sex and age.

Model 3: Adjusted for sex, age, smoking status, CCI, BMI and surgical indication.

Abbreviations: LDI: Lordosis distribution Index, ODI: Oswestry disability Index, CCI: Charleston Comorbidity Index, BMI: Body Mass index.

No interaction was found in either of the adjusted analyses (data not shown).

Repeated analyses with EQ-5D as dependent variable did not reveal any significant difference in the logistic analysis or the MANOVA.

## 4 Discussion

Spinal fusion is increasingly being used for treating lumbar degenerative spine disease causing low back pain, and posterolateral pedicle screws with TLIF has shown to be among the preferred types of surgery.^4^^,^^6^^,^^28^ The effect of surgery assessed by PROMs has previously been discussed in the literature.^5^^,^^29^ However, the effect of lordosis maldistribution on the clinical outcome is not explored, and recent evidence suggest that previous indicators as PI-LL mismatch does not adequately encompass all aspects of how sagittal balance effects the clinical results of surgery.

This longitudinal retrospective cohort study assessed the relation between a relatively new indicator – LDI – and PROMs 12 months after surgery. Surgery showed effect in all PROM data, ODI, VAS back pain, VAS leg pain and EQ-5D, and 54 % of the patients achieved MCID on the primary clinical outcome variable ODI.

The primary outcome of the present study was change in ODI correlated to postoperative LDI. No significant difference between groups was observed when comparing the three different LDI groups. When other PROMs were examined, no significant effect on absolute changes or MCID was observed, neither in the unadjusted or the adjusted analyses.

The sagittal parameters PT and SS after surgery were significantly different between the LDI groups, but the overall effect of surgery on LDI was not significantly different among the groups.

The revision-rate at 12 months follow up was lowest in the normolordotic group, being 11.4 %, compared to 16.7 % in the hypolordotic and 28.6 % in the hyperlordotic group of patients, although the difference was not statistically significant.

Previous evidence has shown that postoperative LDI maldistribution has a higher correlation to Adjacent Segment Disease (ASD),^18^ than PI-LL mismatch.^9^

ASD is related to increased risk of revision and to a negative effect on the overall clinical outcome of surgery.^8^^,^^9^ This could possibly explain the lack of difference in PROMs between LDI groups in our material: i.e., patients with post-operative maldistributed LDI could be subject could have inferior clinical results due to ASD, which ultimately could lead to revision surgery. This is supported by the increased risk of revision in lordotic maldistributed patients.

Adding to this, patients were grouped based on radiographical evaluation at 3 months after surgery and the “LDI- endpoint” after possible revision is not necessarily reflected in the LDI-group of the individual patient.

The effect of surgery on LDI is evidently different between groups, as normolordotic patients tend to stay normolordotic, whereas both hypo- and hyperlordotic LDI tend to get exaggerated. Also, PI-LL is maldistributed in both the hypo- and hyperlordotic patients, while normal in normolordotic patients. Also, an opposed distributive effect of surgery is seen in patients evaluated as hypolordotic and hyperlordotic after operation, as hypolordotic patients undergo respectively negative PT-, positive LL-, and positive SS-changes whereas hyperlordotic vice-versa.

As iatrogenic hypolordosis may predispose later Adult Spinal Deformity, it is crucial to minimize the risk of such iatrogenic hypolordosis.^30^ By using the PI as reference, and subsequently aiming for a similar magnitude of lordosis (PI-LL), radiographic goals and postoperative assessment of lordosis were made possible. However, how to distribute the lordosis, from the lower segments to the upper, remains an unanswered question. Using the normal sagittal shape as reference, the LDI has been proposed as a descriptive tool to assess ratio of the larger lower lordosis to the upper lordosis of less magnitude.^13^^,^^14^

The effect of a postoperative normolordotic spine following surgery for sagittal Adult Spinal Deformity, suggest increased risks of revision surgery due to e.g. proximal junctional failure (PJK) in patients where the postoperative LDI was hypolordotically maldistributed, suggesting a too small lower lordosis. Combined with the established risk of Adult Spinal Deformity due to iatrogenic hypolordosis, we sought to assess if the LDI could be used to identify patients following TLIF surgery that might later develop sagittal spinal deformity. However, no such evidence was apparent in the current patient population, although we did find a slightly lesser rate of revision surgery in the normolordotic group. Further assessment in larger patient samples is warranted before interventional studies.

Some evidence has indicated that postoperative clinical outcome reaches its final level within 24 months postoperatively.^12^ It is thus not possible to observe the expected full effect on PROMs within our 12 months follow up. This also correlates with Fig. 2, where the dynamic changes in radiographical parameters are seen from 3 months to 12 months postoperatively, although this was not examined further in this study. It could be speculated whether a significant difference between groups could be seen if assessed final level. Full radiological assessment at 24 months was, however, not possible due to regional guidelines of only 12 months postoperative follow-up.

The absence of difference in PROMs between LDI groups could additionally be due to the overall wellbeing of the patient population included in the study.

This study has some limitations. The nature of a retrospective study with limited sample size leaves room for residual confounding and both type I and II errors, although the study design encompasses prospectively collected data leaving no room for recall bias. Further studies, preferably multicenter prospective studies with a larger patient material are needed.

Furthermore, we did not stratify according to the specific spinal degenerative disorder leading to the indication of fusion surgery, which could play a role in PROMs at 12 months follow-up, as the effect on clinical outcome of fusion surgery may differ among various spinal degenerative pathologies. The distribution of the preoperative degenerative diagnoses was different between our postoperative LDI-groups. We sought to counter this by completing several multivariate analyses which revealed no difference between groups. We did not find any interaction between the applied variables, e.g., sex, age, smoking status, CCI, BMI or surgical indication.

Furthermore, the goal was to examine the effect of LDI on PROMs rather than the different pathologies examined.

Our follow-up-rate was 58 %, which is rather low. However, a previous drop-out analysis of the DaneSpine Database revealed that the non-responders had a better clinical improvement compared to the responders,^16^ indicating that our loss-to-follow-up does not confound the overall results of the present study. A Nevertheless, a longer observational period of 24 months instead of the present 12 months follow up could infer a change in the revision rate, as the appropriate follow-up-period may be 24 months, as indicated by a previous study.^12^

24 patients did not have sufficient postoperative imaging, and was thus excluded. We tested the demographics of these patients against the included population and found no significant difference, but confounding can not be outruled.

The concept of MCID has also been a topic of discussion.^25^ The cut-off value for our primary outcome measure, ODI, was 10. A higher cut-off value may have led to a diminished number of patients achieving MCID, which could potentially have a significant influence on the results. To counter this, we examined the influence on the absolute changes in PROMs both in unadjusted and adjusted analyses. These analyses on continuous variables did not show a different result.

## 5 Conclusion

In conclusion, we found no statistically significant correlation between postoperative LDI subgroups of normolordotic, hypo- or hyperlordotic patients and the clinical outcome of posterolateral fusion and TLIF surgery for lumbar degenerative spine diseases. A trend towards lower rate of revision surgery in the normolordotic group compared to the hypo- and hyperlordotic group was observed, which is consistent with previous findings in patients undergoing deformity surgery.

## Funding

Benny Dahl is funded by 10.13039/501100007469The Alfred Benzon Foundation.

## CRediT authorship contribution statement

**Anders Schack:** Conceptualization, Data curation, Formal analysis, Investigation, Methodology, Project administration, Writing - original draft. **Tanvir Johanning Bari:** Methodology, Supervision. **Martin Gehrchen:** Writing - review & editing. **Benny Dahl:** Writing - review & editing. **Rachid Bech-Azeddine:** Conceptualization, Methodology, Writing - review & editing.

## Declaration of competing interest

The authors declare that they have no known competing financial interests or personal relationships that could have appeared to influence the work reported in this paper.

## Abbreviations

LDI, Lordosis Distribution Index; TLIF, Transforaminal Lumbar Interbody Fusion; PROMs, Patient Related Outcome Measures; MRI, Magnetic Resonance Imaging; PLF, Posterolateral Fusion; PI-LL, Pelvic Incidence - Lumbar Lordosis; ASD, Adjacent Segment Disease; PJK, Proximal Junctional Failure; CCI, Charleston Comorbidity Index; VAS, Visual Analog Scale; ODI, Oswestry Disability Index; EQ-5D, European Quality of Life - 5 Dimensions Questionnaire; SD, Standard Deviation; IQR, Interquartile Range; ANOVA, Analysis of Variance; MANOVA, Multivariate Analysis of Variance; IRB, Institutional Review Board; BMI, Body Mass Index; MCID, Minimal Clinical Important Difference; SS, Sacral Slope; PT, Pelvic Tilt
